# The effect of chiropractic treatment on infantile colic: study protocol for a single-blind randomized controlled trial

**DOI:** 10.1186/s12998-018-0188-9

**Published:** 2018-06-07

**Authors:** Lise Vilstrup Holm, Dorte Ejg Jarbøl, Henrik Wulff Christensen, Jens Søndergaard, Lise Hestbæk

**Affiliations:** 1 Nordic Institute of Chiropractic and Clinical Biomechanics, University of Southern Denmark, Campusvej 55, DK-5230 Odense M, Denmark; 2 Research Unit of General Practice in Odense, University of Southern Denmark, J.B. Winsløws vej 9A, DK-5000 Odense C, Denmark; 3 Dept. Of Sports Science and Clinical Biomechanics, University of Southern Denmark, Campusvej 55, 5230 Odense C, Denmark

**Keywords:** Randomized controlled trial, Chiropractic treatment, Manual therapy, Infantile colic, Infant, Child

## Abstract

**Background:**

Infantile colic is a common condition during early childhood affecting around one of six newborns. The condition is characterized by inconsolable crying and fussing in otherwise healthy and thriving infants. The most used definition is excessive crying for at least three hours a day for at least three days for at least three weeks. The cause of colic is still unknown although many hypotheses and thereby many different treatment modalities have been investigated. Chiropractic care is used increasingly in treatment of infants, including for infantile colic, although the evidence worldwide is sparse. A randomized, controlled trial was designed to evaluate the effect of chiropractic treatment on infantile colic. This paper describes the protocol as well as results from a pilot study examining the acceptability and feasibility of the intervention.

**Method:**

The study is designed as a single-blind randomized, controlled trial. The invited families are residents on the Island of Funen and information about the project is distributed from the maternity wards and health visitors. Children at the age of 2–14 weeks with unexplained excessive crying are screened for eligibility and recruited by the primary investigator through home visits. Eligible children are then randomized to chiropractic treatment or control. All children attend in the chiropractor clinic two times a week for two weeks. The parents are unaware of their child’s allocation during the project period. The primary outcome measure is change in daily hours of crying based on the parental diaries.

The study intends to include 200 children, and the intervention has, during a pilot study, been found acceptable and feasible among families with newborns.

**Discussion:**

In a single-blind randomized controlled design we will evaluate the effectiveness of chiropractic treatment on infantile colic. The study will contribute to determine the effect of chiropractic treatment on infantile colic in an area where limited evidence exists. Furthermore, the study aims to explore if subgroups of children with suspected musculoskeletal problems will benefit more from the intervention than others. If they obtain better results, this could imply the need for stratified care.

**Trial registration:**

Clinicaltrials.gov and Identifier: NCT02595515 (registered 2 November 2015).

**Electronic supplementary material:**

The online version of this article (10.1186/s12998-018-0188-9) contains supplementary material, which is available to authorized users.

## Background

Infantile colic is a common condition during early childhood affecting on average one of six newborns. The condition is characterized by inconsolable crying and fussing in otherwise healthy thriving infants. The most used definition is excessive crying for at least three hours a day for at least three days a week for at least three weeks. The babies are between two and four months, with a mean onset of colic at two weeks and an average duration of symptoms of four to five months [1, 2].

Although the symptoms in most cases cease at the age of four to five months and very rarely indicate serious underlying disease, unexplained crying is a common cause for seeking professional medical advice [3]. This may reflect the burden and stress experienced by the families of a colicky child [4, 5]. Having a colicky child has been associated with higher risk of maternal postpartum depressive symptoms and a high level of distress even after the colic has stopped [6–8]. In the extreme, inconsolable crying can lead to child abuse, and has been associated with baby shaken syndrome [9, 10]. Furthermore, even though colic is considered a ‘benign and self-limiting’ condition, an increasing number of studies indicate there may be sequelae to this condition, including later developmental and behavioral problems, such as sleeping disorders, sensory processing abilities, lack of concentration, hyperactivity and temper tantrums [6, 11–13].

The aetiology of infantile colic still remains to be established. The original meaning of the word ‘colic’ is ‘large intestine’ which implies that a cause in the digestive or gastrointestinal system, resulting in painful contractions of the gut, has traditionally been suspected. Hypotheses regarding the cause of excessive crying have also frequently involved the gastrointestinal system, e.g. immaturity [14], allergy towards cow’s milk [15], transient lactose intolerance [16], intestinal microflora [17] and motility dysfunction [18]. Other hypotheses include disturbances in the parent-child relationship [19], factors related to the pregnancy and birth [20, 21] or merely the extreme of normal crying [22]. Based on the different aetiological hypotheses many different interventions have been performed but the results are ambiguous, and imply that colic may have a multifactorial aetiology with the need for a more stratified treatment approach [23–26].

Another commonly believed theory is that these children cry because of pain that origins from the musculoskeletal system [27], and thus manipulative therapy could be indicated as part of the solution.

Worldwide, children, including infants, are increasingly diagnosed and treated in chiropractic clinics, [28, 29], a tendency also seen in Denmark where chiropractic visits for infants have more than doubled over the last ten years with infantile colic or excessive crying being the main cause [30, 31]. In contrast to this, the effect of chiropractic treatment on infantile colic has only been investigated in few randomized, controlled trials, and the results are ambiguous. A Cochrane review regarding manipulative therapies for infantile colic identified six randomized trials representing a total of 325 infants [27]. Of the six studies, five showed a positive effect on daily hours of crying, and one found no difference when compared to the natural cause of infantile colic. However, in general there were methodological weaknesses, since the studies were generally small, and furthermore in most studies parents were aware if the child was treated or not, which increases the risk of a placebo effect in the intervention group. Since chiropractic treatment of infantile colic is widely used, there is a need to investigate the effectiveness on the condition in a larger scale study with parental blinding [27].

A randomized controlled trial was designed to evaluate the effect of chiropractic treatment on crying time and other symptoms associated with infantile colic. Furthermore, the study aims to explore if subgroups of children with suspected musculoskeletal problems will benefit more from the intervention than others. This article will describe the protocol as well as the results from a pilot study examining the acceptability and feasibility of the intervention.

## Method

### Study design

Multicenter single-blind randomized controlled trial.

### Study setting and participants (Fig. 1; Additional file 1)

#### Recruitment

The invited families are residents on the Island of Funen (approx. 500.000 inhabitants). Information about the existence of the project is given by: 1) health visitors, 2) general practitioners, 3) written information about the study in a folder given to all women that has given birth at Odense University Hospital, 4) the chiropractic clinics participating in the study and 5) advertisements in local media and on Facebook. Parents are encouraged to contact the primary investigator (PI) by telephone, where she will screen the child for in- and exclusion criteria. If inclusion criteria seem to be fulfilled, and the parents agree to participate, a more thorough interview is arranged to take place in the home of the family.

**Fig. 1:**
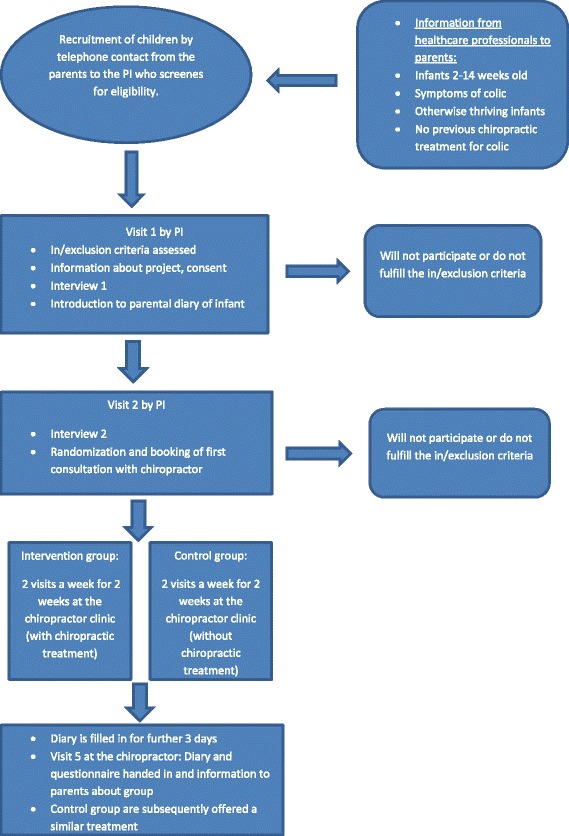
Study flow chart and time lime for participants

#### Visit 1

At the first visit, the PI, who is a medical doctor, assesses the child for eligibility and provides oral and written information (Additional file 2) regarding the project.

At the first visit the PI furthermore completes an interview-based questionnaire (Additional file 3) with the purpose to collect information about baseline variables and potential clinical predictors and confounders. The choice of variables was based on knowledge from existing literature [6–8, 19–21, 23, 27, 32] and focus group discussions with chiropractors and health visitors. Questions concerns the child’s crying pattern, birth weight and length, current weight and length, feeding mode and pattern, burps, regurgitation, bowel movements, anamnestic and objective signs of musculoskeletal problems (e.g. favorite side), previous treatment of the colic or other condition/disease, the mother’s health and use of medication, the parents’ level of serious mental stress during pregnancy and after the birth, mental stress of the parents in everyday life, educational level and cohabitation status of the parents, smoking status of the parents, siblings with history of colic, the parents’ belief regarding the effect of chiropractic treatment on colic, and selected factors related to pregnancy and birth. With consent of the parents pregnancy and birth related factors will also be collected in the mother’s hospital record, e.g. mother diagnosed with depression, gestational age at birth, durations of the different stages in birth, abnormal presentation in the birth canal and interventions in birth.

If the parents’ consent to participate with their child (Additional file 4), they are introduced to a diary for use during the study (Additional file 5). This diary represents the parents’ structured notes of the child’s behavior, a validated method that has proved to be reliable as an objective recording of the child’s behavior and symptoms [33]. The parents are thoroughly instructed to record the child’s behavior throughout every 24 h during the whole project period. ‘Time rulers’ representing every 24 h divided in time intervals of fifteen minutes are filled out with different symbols representing the child’s behavior, including the amount of inconsolable crying (crying without an obvious reason that cannot be comforted by any attempt of the parents), the time the child needs to be held and rocked to limit crying (crying without no obvious reason that is only partly and briefly limited if the child is constantly held/rocked), the time the child is awake and content, and the time spent sleeping, feeding patterns and bowel movements. Consolable crying with an obvious reason that is easily comforted as e.g. hunger or a soiled diaper is registered as ‘awake and content’. To limit recall bias and make the registration as precise as possible the parents are advised to fill in the diary several times a day. Baseline registration of the crying pattern is done for at least three days whereupon a second visit from the PI is scheduled.

#### Visit 2

At the second visit, any difficulties filling out the daily diary is identified and the parents’ assessment of the crying pattern is evaluated in a second interview-based questionnaire (Additional file 6). Eligible children are then randomized, and an appointment is booked in one of the participating chiropractic clinics (parents’ choice). Four chiropractic clinics with seven chiropractors participate in the study, and all the chiropractors have a special interest and experience in pediatric practice. The treating chiropractors have been working from nine years to more than thirty years in the field.

### Inclusion criteria

Infants aged 2 to 14 weeks with symptoms of infantile colic defined as otherwise healthy thriving infants with episodes of excessive crying that last at least three hours a day, for at least three days a week in the past two weeks. During the crying episodes the child cannot, or only briefly and partly, be comforted. The type and duration of the crying episodes is assessed by the parents after thorough instruction by the PI as described above (*visit 1*). Except from these crying episodes, the child must show normal development and gain at least 150 g a week (it is though considered normal that the child in the first week of life loses up to 10% of the birth weight, which is hereafter regained).

### Exclusion criteria

The child cannot have symptoms of disease, suffer from a current disease or have sequelae from a former disease. The child cannot have contraindications for chiropractic treatment, e.g. certain congenital malformations, or have received chiropractic treatment for colic previously. Concomitant treatment for colic (e.g. reflexology) is not permitted during the project period.

### Randomization and blinding

The allocation occurs through a 1:1 ratio by a predetermined restricted randomization scheme. The participants are stratified according to their age at enrollment (2–6 weeks; 7–10 weeks; 11–14 weeks) and the treating chiropractor. The eligible participants are randomly assigned in blocks of 4 to 6, according to computer-generated random numbers, to be enrolled in either the intervention or control group.

The computer generated block randomization was administered by a research assistant, not otherwise involved in the outcome measurements or the intervention. The research assistant then wrote the consecutive letters A and B on separate pieces of paper and then placed them in sealed opaque envelopes, which were given to each chiropractor. To take the above mentioned stratification into account the procedure was repeated and a different list of group allocation was made for each age level and each chiropractor in the study. When a child is randomized and an appointment booked, the PI informs the clinic of the next randomization number for that specific chiropractor and age group (which is a coded number not revealing group allocation). Only the chiropractor has the list that reveals if that certain randomization number means allocation to intervention or control group.

Hence, the intervention in this trial makes blinding of care providers impossible. The parents are blinded to their child’s allocation group during the project period. To uphold blinding of the randomization, all parents leave the chiropractor’s consultation room for a few minutes after history taking and examination, while the treatment is (or is not) carried out. For the analyses, the coding of intervention and control groups will be concealed from the PI and the statisticians performing the analyses. The randomization code will not be broken until the analyses are completed.

### Intervention

The study is pragmatic and assesses the effect of the treatment the clinician finds indicated, rather than a standard treatment. Therefore the study does not investigate a specific manual treatment, but investigates the whole chiropractic intervention with individual attention to the children’s potential biomechanical dysfunctions, as described below.

All children attend the chiropractor clinic two times a week for two weeks. After the fourth visit the parents continue their registration in the diary as an ‘after registration’ for at least three days, and fill out the final questionnaire including questions regarding the status of their child’s colic, bowel movements, burps, regurgitation and their belief regarding group allocation (Additional file 7). Hereafter an information visit takes place at the chiropractic clinic, where the parents hand in the completed diary and questionnaire and are informed whether their child has been in the treatment or control group. Children in the control group are then offered a similar treatment free of charge as the intervention group, if symptoms have not subsided, a strategy to improve adherence to the study.

#### The control group receives

- Medical history
- Examination without motion palpation of the joints, as this could approach mobilization. Examination includes undressing the child and observing if there are notable asymmetries of limb orientation and development, and distortions of the head, spine and body shape that could indicate a biomechanical dysfunction (related to muscles, ligaments and/or joints). Furthermore, the examination includes a mental and neurological status
- Based on the medical history and examination the clinician estimates whether the child’s symptoms are likely to be related to a biomechanical dysfunction
- Pragmatic advice such as cycling with the child’s legs and changing the position of the child’s head from one nap to the next
- At the last visit, the chiropractor assesses if the child possibly can benefit from a similar treatment as given in the intervention group.

#### The intervention group receives

- Medical history
- Full examination, including motion palpation of the joints. To diagnose a biomechanical dysfunction examination includes static and movement palpation, observation of notable asymmetries of limb orientation and development, and distortions of the head, spine and body shape as well as exploration of muscular and ligamentous tonus and movement restrictions in articulations. Furthermore, mental and neurological status.
- Based on the medical history and examination the clinician estimates whether the child’s symptoms are likely to be related to a biomechanical dysfunction. This assessment is done before motion palpation of the joints and reassessed again after the motion palpation.
- Manual treatment will include manipulation or mobilization of the spine and/or the extremities as indicated by the child’s potential biomechanical dysfunctions, including movement restriction, tenderness or an obvious asymmetry in the muscles or joints. If no biomechanical dysfunction is detected mobilization is carried out where all the joints in the spine are lead through their normal range of movement.
- Specific advice directed towards any biomechanical dysfunction, and exercises that supports the effect of the manual treatment
- At the last visit the chiropractor assesses if the child’s course of treatment is completed or whether continued treatment is indicated

The manual therapy in the intervention group is thus administered when there is biomechanical dysfunction in one or more joints which the treating clinician relates to the child’s symptoms. In general, the treatment technique for restricted movements in joints in this age group is a very light short-term pressure with fingertips and gentle massage in case of hypertonic muscles.

The frequency and content of treatments is intended to resemble pragmatic daily clinical practice in order to optimize the treatments by individualization and to make the results generalizable.

### Outcome measures

The primary outcome measure is change in daily hours of crying (inconsolable crying and the time the child needs to be held and rocked to limit crying) based on the parental diaries (before and after treatment).

Secondary outcomes are based on information from parental diaries and the final questionnaire filled out by the parents and will include:

- Sleeping hours per day (change in mean daily hours of sleeping before and after treatment)
- Awake and content (change in mean daily hours of an awake and content baby before and after treatment)
- General Perceived Effect (colic has stopped/decreased/unchanged/increased)
- Bowel movements (more often/more rarely; easier/more difficult)
- Burps (easier/more difficult)
- Regurgitation (more/less)

#### Potential clinical predictors

- Musculoskeletal problems as based on information from interview-based questionnaires and the chiropractors’ examination defined by favorite side during feeding or sleep, does not want to lie in abdominal position, difficulties dressing/undressing the child, objective signs as asymmetric gluteal folds/hips/knees/tonus in back musculature or skew position/C-curve and dichotomized into yes/no
- Factors related to pregnancy or birth e.g. complications related to pregnancy, induction of labor, duration of birth and different stages in birth, abnormal presentation in birth canal, interventions in birth such as vacuum assisted delivery and cesarean. This information will be drawn from interview-based questionnaires and supplemented if needed from hospital records and dichotomized into yes/no or categorized into appropriate categories.

Potential confounders include parental highest attained education, cohabitation status, smoking status of parents, birthweight, siblings with history of colic, stress in everyday life, severe stress related episodes during pregnancy or birth (e.g. severe illness or death among family members or close friends), mental illness of mother (e.g. depression). This information is obtained from the interview-based questionnaires. Other potential confounders are age and treating chiropractor which is accounted for in the stratified block randomization.

### Sample size

Based on data from the pilot study we observed a mean reduction in hours of crying in the control group of 2.5 h per day before and after treatment with a standard deviation of 2.5 h. Based on group discussions in the research group to obtain a clinically satisfactory result, we therefore wish that children in the intervention group will have a reduction of one additional hour, resulting in a reduction of 3.5 crying hours. With an assumption of a common standard deviation of 2.5, a level of significance on 0.05 and a power of 80%, this result in a sample size of 200 children distributed with 100 in each group.

### Statistical analyses

For the primary analyses the main endpoint is change in mean number of hours/24 h spent with excessive crying before and after the two weeks treatment period, comparing treated and not treated children (mean difference in hours of crying with standard deviations and *p*-value for difference between the groups using a two-sample t-test). Change in mean hours before and after equals 3–4 days before treatment and 3–4 days after treatment. All analyses will be performed by intention-to-treat principles and no data will be imputed.

To evaluate the independent effect of treatment, multiple linear regression analyses of the change in crying hours will be performed including baseline values of the crying hours, randomization group and stratification factors as independent variables.

Secondary outcomes will be analyzed similarly. Furthermore, a secondary repeated measures linear regression analysis will evaluate change over time with regard to time spent crying, sleeping, and time being awake and content.

In a secondary explorative analysis it will be evaluated if subgroups of children with suspected musculoskeletal problems will benefit more from the intervention than others, and all analyses will be repeated stratified for presence of musculoskeletal problems. This hypothesis is based on the theory that the children cry because of pain that origins from the musculoskeletal system [27]. Furthermore, existing literature suggest there may be a relationship between complicated births and infantile colic [20, 34], and one theory is that complicated births may induce musculoskeletal problems [23, 27]. We therefore hypothesized that a potential beneficial treatment effect of chiropractic care may be greater for the children born by a complicated birth when compared to the children born at an uncomplicated birth. Therefore, similarly to presence of musculoskeletal problems, analyses will be repeated stratified for elected factors related to birth. These analyses will be explorative in nature, but could help to identify potential clinical predictors of treatment effects to be incorporated in future studies. Analyses will also be performed on the different age and chiropractor strata.

In the stratified secondary analyses, clinical relevant confounders will be included in the analyses in consideration of the number of individuals in each stratum. These analyses will only be performed when there are a sufficient number of individuals in the strata.

All results will be published in relevant peer reviewed scientific journals and subsequently in the Danish media.

### Ethics

No serious or lasting side effects have ever been reported in infants following the types of treatment used in this trial and no compensations claims have ever been made for this this age group in Denmark [35]. Because there is no experimental treatment involved, but only treatments, which are usually performed in clinical practice, no interim analyses are made and therefore no data monitoring committee is needed. Furthermore, no standardized treatment in the area exists that the child is withdrawn from.

All parents have given written informed consent for their child to participate in the study and for collection of information from hospital files (Additional file 4). Participation in this study is voluntary, and the parents can withdraw their child from the study at any time with no negative consequences for the child. Parents are advised to immediately contact the chiropractor (who can contact the PI if relevant) if they suspect any serious adverse effect during the project period. In any case of discontinuation, the child’s allocation group will be revealed for the parents. All cases of discontinuations/withdrawals will be registered with reason for withdrawal.

The study has been approved by The Regional Committee on Health Research Ethics (S-2015001), and data are being handled by a data manager at NIKKB according to regulations by the Danish Data Protection Agency. The Regional Committee on Health Research Ethics reviews an annual safety report and any amendments made in the protocol or the documents used in the trial.

### Results from the pilot study

A pilot study was carried out from September 2015 to March 2016 with the main purpose to examine the acceptability and feasibility of the intervention. Within the first months, the study was introduced to health care professionals (health visitors, midwifes, general practitioners). Furthermore, advertisements were made in local media (interviews with PI about the project in local papers, television and radio). At the end of the pilot study it was clear, that the majority of the participants in the study were referred by the health visitors. Therefore, the contact to health visitors has been intensified with regular newsletters and presentations about the study.

Within the inclusion period of 5 months, 60 parents contacted the PI. Around half of these fulfilled the inclusion criteria and accepted to participate. The main reasons for not participating were parental refusal (due to the risk of being randomized to the control group or too time consuming transportation to the chiropractic clinic), the child had already been treated for colic by a chiropractor, or the child was too old. As a consequence, the age criteria were extended from 2 to 10 weeks to 2–14 weeks in the main project and an additional chiropractic clinic was included to accommodate participants geographically. In order to take into account factors that may influence outcome (increasing age span of children and increasing number of treating chiropractors) the randomization scheme was optimized so randomization in the main study was stratified according to child’s age and treating chiropractor. All the changes were reported as amendments to the protocol in April 2016 and approved by The Regional Committee on Health Research Ethics.

Furthermore, in order to reach and inform the parents about the project before they possibly contacted a chiropractor on their own initiative, health visitors were asked to inform parents about the existence of the project already at their first home visit when the baby was 1–2 weeks old.

Based on the pilot study it was concluded that the study was generally well accepted among new parents and health care professionals and it was estimated that the time frame for the main project would be two to three years.

## Discussion

The four main strengths of this study are 1) the large size, 2) the possibilities for subgroup analyses, 3) the blinding of the parents and 4) individualized treatment. This will meet the shortcomings of previous studies and make potential results generalizable. Furthermore, a major strength of the study is that a pilot phase evaluated feasibility and acceptability of the study and gave the opportunity to optimize several important factors.

Limitations include that blinding of practitioners was not possible due to the nature of the study. Furthermore, a longer follow-up period to measure potential long-term effects of the treatment would have been beneficial, however the experiences from the pilot study showed us that the study had an acceptable length that should not be prolonged. The main cause for this being that the parents often were very stressed due to their child’s colic and had to endure the uncertainty about their child’s treatment throughout the project period. Two weeks seemed to be the maximum period, parents were willing to accept this. Another potential limitation is that there might be a potential reduction of effect size if there are too few children in the treatment group who actually have biomechanical problems. Hopefully, the randomization will distribute children with and without biomechanical problems evenly among the two groups, and furthermore, the secondary analyses can limit the impact of this limitation.

The study will contribute to establish the effect of chiropractic treatment on infantile colic in an area where limited evidence exists [27]. An increasing number of infants are treated in chiropractic clinics, and the results of the study will be highly relevant for all new parents seeking chiropractic care for their child’s colic. Furthermore, the results will support healthcare professionals in guidance of parents on an evidence based level, which does not exist today. Since associations between infantile colic and both short and long-term consequences for the child and family have been shown [6–8], proper treatment at an early stage might actually present as primary prevention of these disorders. Therefore, a potential positive effect of chiropractic treatment on infantile colic may benefit both individuals and society considerably. Furthermore, serious side effects of chiropractic care in infants have not been reported, and therefore a potential beneficial effect is likely to exceed potential risks [29, 35].

Infantile colic appear to have a multifactorial etiology and it may be unlikely to find one treatment that will fit all [24, 25, 27]. Therefore there is a need to explore if certain subgroups will benefit more from a specific treatment. If confirmed, this could indicate a causal relation and form the basis for a more stratified care that does not exist today.

### Trial status

Patient recruitment is ongoing and will continue until 200 participants have been included or approximately  ultimo 2018.

## Additional files

Additional file 1: SPIRIT diagram for the schedule of enrolment, intervention, and assessments for each participant. (DOC 48.5 kb)

Additional file 2: Participant information sheet. (DOCX 23 kb)

Additional file 3: Interview-based questionnaire 1. (DOC 54 kb)

Additional file 4: Consent form. (DOC 34 kb)

Additional file 5: Parental diary of infant behavior. (DOCX 17 kb)

Additional file 6: Interview-based questionnaire 2. (DOC 28 kb)

Additional file 7: Parents final questionnaire. (DOC 35 kb)

## Abbreviation

PI: Primary Investigator
