# Supplementary material for: The effect of chiropractic treatment on infantile colic: study protocol for a single-blind randomized controlled trial
Source: Chiropr Man Therap. 2018 Jun 7;26:17. doi: 10.1186/s12998-018-0188-9 (PMC5991429; doi:10.1186/s12998-018-0188-9)
Supplement: Supplementary file 1 — SPIRIT diagram for the schedule of enrolment, intervention, and assessments for each participant. (DOC 48.5 kb) [file 12998_2018_188_MOESM1_ESM.doc]

Figure 2. SPIRIT diagram for the schedule of enrolment, intervention, and assessments for each participant. Compliments Figure 1.

|  | **Enrolment** | **Allocation** | **Post-allocation** | |
| --- | --- | --- | --- | --- |
| **TIMEPOINT** | **Minimum 3 days before allocation** | ***t=0*** | ***t_1=_day 1-14*** | ***t_2=_day 15-17*** |
| **ENROLMENT:** |  |  |  |  |
| **Eligibility screen** | X |  |  |  |
| **Informed consent** | X |  |  |  |
| **Allocation** |  | X |  |  |
| **INTERVENTION*:** |  |  |  |  |
| **Intervention** |  |  | X |  |
| **Control** |  |  | X |  |
| **ASSESSMENTS:** |  |  |  |  |
| ***Baseline variables***** | X |  |  |  |
| ***Outcome variables***** |  |  | X | X |
| ***Other data variables (clinical predictors and confounders***** | X |  |  |  |

*See description of the intervention in main protocol

** See description of collected variables in main protocol
