# Supplementary material for: The effect of chiropractic treatment on infantile colic: study protocol for a single-blind randomized controlled trial
Source: Chiropr Man Therap. 2018 Jun 7;26:17. doi: 10.1186/s12998-018-0188-9 (PMC5991429; doi:10.1186/s12998-018-0188-9)
Supplement: Supplementary file 2 — Participant information sheet. (DOCX 23 kb) [file 12998_2018_188_MOESM2_ESM.docx]

**Participant information regarding participation in a scientific study**

Title of the study: The effect of chiropractic treatment on infantile colic

We would like to, with your consent, to invite your child to participate in a scientific study carried out by the Nordic Institute of Chiropractic and Clinical Biomechanics and the Research Unit for General Practice, University of Southern Denmark.

Before you decide whether your child can participate in the study, you must fully understand what the study is about and why we conduct the study. We therefore ask you to read this participant information thoroughly.

You will be invited to an interview as part of the study, where this participant information will be elaborated. Ask questions if anything you reed is not clear or you would like more information. If possible we would like both parents to be present.

If you decide to let your child participate in the study, we will ask you to sign a letter of consent on behalf of your child. Remember that you are entitled to consideration before you decide whether to sign the statement of consent.

It is voluntary to participate in the study. You may, at any time and without giving a reason withdraw your consent.

**Purpose of the study**

Chiropractors have good experience with treatment of infants, parents seek the treatment and annually several thousand infants are treated in the Danish chiropractic clinics. It is therefore important to determine whether it has an effect and if so, to identify the children who are most likely to benefit from the treatment.

Chiropractic treatment is concerned with diagnoses and treatment of musculoskeletal problems. If chiropractic treatment has an effect on excessive crying, it is therefore reasonable to assume that the effect will be found in those children whose cry is caused by a problem in the musculoskeletal system.

Therefore, the primary purpose of the study is to investigate the effect of chiropractic treatment on infantile colic. In addition, we will investigate whether children with suspected problems in muscles and joints (musculoskeletal problems) benefit more from chiropractic treatment than others.

**Schedule of the study**

The collection of data from patients has started autumn 2015 and will continue for approximately three years. Data must be collected from 200 infants.

For you, the schedule will be:

When you have contacted the project manager, you will within a few days be offered a visit in your own home. The project manager will inform you about the study and instruct how you to fill out the forms that will be used for the observation of the child.

You will be asked if the project manager can obtain relevant information about the pregnancy and birth from the mother’s hospital record. More specifically, this refers to diagnoses and treatment of complications related to pregnancy including preeclampsia, elevated blood pressure, threating premature birth, bleeding, and depression. In addition, factors related to the birth including gestational age at birth date, abnormal presentation in the birth canal (e.g. breech, shoulder presentation), intervention in the birth (e.g. induction of labor with medicine or other medications during birth such as epidural anesthesia, vacuum-assisted delivery, caesarean section), other complications related to birth (sphincter rupture, bleeding that exceeds 500 ml, asphyxia). This information will be included in analyses that evaluates whether pregnancy and childbirth conditions may be associated with colic.

If the project manager estimates that your child fulfill the criteria for participation in the project and you agree that your child can participate the project, you will be instructed to complete an observation schedule for three days in order to record the crying pattern for your child. The project manager evaluates the schedule after the three days, and you will then have an appointment at the chiropractor. Several chiropractic clinics are connected to the project in your area, and you can choose the one that suits you best. The chiropractor will examine the child and evaluate if there are any reasons why the child cannot get chiropractic treatment. If this is not the case, your child will be included in the project. Following a principle of coincidence, half of the children will receive treatment and the other half will not receive treatment. Since you as a parent should observe the child at home and evaluate the effect of treatment, you cannot know if the child is being treated or not. Therefore, both parents must leave the treatment room while the treatment is given (or not given). The chiropractic treatment will primarily consist of treatment of the muscles and joints of the body. The treatment is performed with a light finger pressure (mobilization) around the spine and possibly hips and shoulders. There are either four visit with treatment or four visits without treatment for two weeks (depending on which group your child is in), after which treatment is terminated. If your child has been in the untreated group, you will then be offered to receive active treatment for the following two weeks. All treatments are free for you. Upon completion of treatment, we will ask you to answer some questions about the process and your overall assessment of changes in the child's well-being.

**Benefits**

There is currently no treatment that has a well-documented effect on infantile colic. Chiropractic treatment is one of the methods the parents usually seek.

Considering the prevalence of colic and the possible consequences for both the child and the family, it is obvious to work systematically to find a treatment for these children and their families. When so many children receive chiropractic treatment for colic, it is important to determine whether it has an effect and if so, to identify the children who possibly will benefit the most from the treatment. By participating in the project you help to clarify this issue for the many families affected by colic. Furthermore, you also get the opportunity to receive this treatment for your child under very controlled conditions and free of charge for you.

**Side effects and risks**

No serious or lasting side effects have ever been reported in infants following the type of treatment used in this trial and no compensations claims have ever been made for this this age group in Denmark. There may be risks of the attempt, which we do not yet know. We therefore ask you to tell if you experience problems with your child's health while the experiment is ongoing. If we notice any unexpected side effects, you will of course be informed immediately, and you will have to decide if you wish to continue in the trial.

**Alternative treatment options**

There is currently no well documented treatment for infantile colic.

**Exclusion from the study**

If your child during the project period receives other treatment, we will have to exclude him/her from the study.

**Financial support**

The project is started as an initiative of the Nordic Institute of Chiropractic and Clinical Biomechanics, University of Southern Denmark, Odense in collaboration with the Research Unit for General Practice in Odense, University of Southern Denmark. The project is supported financially by the Danish Chiropractic Fund, The Fund for General Practice and the European Centre for Chiropractic Research Excellence.

**Publication of the study results**

The results of the study will be published in international scientific journals and then in the Danish media

We hope that with this information you have sufficient insight into what it means to participate in the study and that you feel prepared to make the decision regarding participation. We also ask you to read the attached material "Rights of subjects in a biomedical research project".

If you want to know more about the study, please contact Lise Vilstrup Holm, Research Unit for General Practice, J.B Winsløvs Vej 9A, 5000 Odense C, telephone: +45 26278015, e-mail: lholm@health.sdu.dk.

Kind regards

Lise Vilstrup Holm, Project manager, MD, PhD

Research Unit of General Practice in Odense and Nordic Institute of Chiropractic and Clinical Biomechanics, University of Southern Denmark
