# Supplementary material for: The effect of chiropractic treatment on infantile colic: study protocol for a single-blind randomized controlled trial
Source: Chiropr Man Therap. 2018 Jun 7;26:17. doi: 10.1186/s12998-018-0188-9 (PMC5991429; doi:10.1186/s12998-018-0188-9)
Supplement: Supplementary file 3 — Interview-based questionnaire 1. (DOC 54 kb) [file 12998_2018_188_MOESM3_ESM.doc]

Appendix 2

*Interview 1*

*Filled out by PI at first visit*

**Interview with parents**

1. **Date:** _______________
2. **Idenfication number of child:**
3. **Date of birth for child:**
4. **Sex of child:** 🞎 boy 🞎 girl
5. **Weight and length of child at birth:**

1. **Current weight and length of child:**
2. **Date of last weight and length:**
3. **Age of child at debut of colic:**
4. **Average number of hours with colic per day:**
5. **If number of hours with colic varies from day to day, state here the extremes:** minimum: maximum: __________
6. **The colic occurs in so short episodes, so the number of hours is difficult to assess.** 🞎 Yes 🞎 No
7. **Typical number of colic episodes per day:**
8. **If number of episodes with colic varies from day to day, state here the extremes**:
    minimum: maksimum: __________
9. **Mark the time spans, where the colic normally occurs:**🞎 06-12 🞎 12-18 🞎 18-24 🞎 24-06
10. **Typical number of days with colic per week:**

**If number of days with colic varies a lot from one week till the next, state here the extremes:**

1. minimum: maximum: __________
2. **The colic has the last week been:**
    🞎 Increasing 🞎 Unaltered 🞎 Decreasing
3. **The child’s behavior during the colic episodes:** Yes No Shortly
   1. The colic cry is easy to distinguish from crying of other reason 🞎 🞎
   2. The child ’fights the air’ with hands 🞎 🞎
   3. The child flexes or curls the legs 🞎 🞎
   4. The child stretches the legs and bends backwards 🞎 🞎
   5. The child has difficulty passing wind/discomfort on bowel movement 🞎 🞎
   6. The child farts a lot

……………………………………………………………………………………….🞎 🞎

- 1. The child often defecates in connection with colic 🞎 🞎
  2. The child can be comforted by feeding 🞎 🞎 🞎
  3. The child can be comforted with a dummy 🞎 🞎 🞎
  4. The child can be comforted by being picked up 🞎 🞎 🞎
  5. The child needs constant cuddeling ..…..………………………………………………………….🞎 🞎
  6. The child is best comforted by being constantly rocked (cradled)……..………………. 🞎 🞎
  7. The child is best comforted lying on abdomen 🞎 🞎
  8. The child can be comforted by use of white noise…………………………………………………… 🞎 🞎
  9. The child appears in pain…………….…………...……………………………………………….🞎 🞎

1. **The child has following signs to indicate a dysfunction in the musculoskeletal system:**

(if yes to ≥ 1, the child is placed in the group of suspected dysfunction in the musculoskeletal system)

Is the child upset when placed on stomach 🞎 yes 🞎 no

Difficulty dressing the child 🞎 yes 🞎 no Change from happy to crying instantly 🞎 yes 🞎 no

Can the child be in deep consistent sleep 🞎 yes 🞎 no

Is the child tense during feeding 🞎 yes 🞎 no

Favorite side - feeding 🞎 yes 🞎 no

Favorite side - sleep.……………………………………………………🞎 yes 🞎 no

1. **How is the child nourished?**
   1. solely breast feeding 🞎
   2. breast and infant formula 🞎
   3. solely infant formula 🞎
   4. other 🞎
2. **Typical amount of feedings per day:**
3. **How often the child has defecation?**
4. **Is the child more comfortable after defecation?** 🞎 yes 🞎 no 🞎 shortly
5. **Does the child easily burp?** 🞎 yes 🞎 no
6. **Does the child regurgitas a lot?** 🞎 yes 🞎 no
7. **Does the child have many hiccups?** 🞎 yes 🞎 no
8. **Has the colic been treated before?** 🞎 yes 🞎 no

a.If yes, how?

change in feeding 🞎

sugar water 🞎

medical treatment 🞎

reflexology….………………………………………………….. 🞎

other 🞎

a.Result of treatment

better 🞎

unchanged 🞎

worse 🞎

1. **Has the child besides the colic had other condition or an illness,
   which demanded treatment?**  🞎 yes 🞎 yes
   1. If yes, which?
   2. What kind of treatment?
2. **Were there any complications during pregnancy?** 🞎 yes 🞎 no

a.If yes, which?

Preeclampsia 🞎

blodtryksforhøjelse 🞎

blødninger 🞎

depression 🞎

andet 🞎

1. **Was the child born full term?** 🞎 yes 🞎 no
   1. If no, weeks before/after
2. **The delivery:**
   1. Spontaneously 🞎
   2. Induced 🞎
   3. Medicine to induce labour 🞎
   4. Vacuum-assisted delivery 🞎
   5. Cesarean section 🞎
   6. Abnormal presentation. 🞎
   7. Sphincter rupture 🞎
   8. Other complications 🞎

1. **Were the parents during pregnancy exposed to serious negative events (deaths in family or close friends, serious illness in family or close friends, unemployment, bankruptcy or similar)?**
    🞎 yes 🞎 no
   1. If yes, which?
2. **Have the parents since the birth been exposed to serious events (deaths in family or close friends, serious illness in family or close friends, unemployment, bankruptcy or similar)?**
    🞎 yes 🞎 no
   1. If yes, which?
3. **The mother: Do you feel stressed in everyday life?**

yes, often 🞎

yes, sometimes 🞎

No, almost never 🞎

Don’t know 🞎

Uoplyst 🞎

1. **The father: Do you feel stressed in everyday life?**

Yes, often 🞎

Yes, sometimes 🞎

No, almost never 🞎

Don’t know 🞎

Not indicated 🞎

1. **Do the parents believe that chiropractic treatment can relieve the child’s symptoms?**
    🞎 yes 🞎 no 🞎 dont know
2. **Does the child have siblings?**

🞎 yes 🞎 no

1. **Siblings who as infants had symptoms of colic?**
    🞎 yes 🞎 no 🞎 dont know
2. **Do the parents smoke?**
    🞎 yes 🞎 no
3. **Does the mother have physical or mental illness?**
    🞎 yes 🞎 no
4. **Is the mother prescribed medication?**
    🞎 yes 🞎 no
5. **Highest attained education of mother?**

Primary and lower secondary school 🞎

Upper secondary school 🞎

Skilled worker 🞎

Higher education with medium length 🞎

Higher education (beyond bachlor) 🞎

Student 🞎

**39. Highest attained education of father?**

Primary and lower secondary school 🞎

Upper secondary school 🞎

Skilled worker 🞎

Higher education with medium length 🞎

Higher education (beyond bachelor) 🞎

Student 🞎

**40. Cohabitation status:** Married/cohabiting 🞎

Living alone 🞎

**41. How did you learn about the project? _________________________**

**Examination by principal investigator**

**Weight gain satisfactory** 🞎 yes 🞎 no

**The child’s development assessed normal** 🞎 yes 🞎 no

**The child has following signs that indicate dysfunction in the musculoskeletal system:**

(if yes to ≥ 1, the child is placed in the group of suspected dysfunction in the musculoskeletal system)

Asymmetric gluteal folds 🞎 yes 🞎 no Asymmetric hips 🞎 yes 🞎 no

Asymmetric knees 🞎 yes 🞎 no

Skew position in spine (C-curve) 🞎 yes 🞎 no Asymmetric tonus in back musculature 🞎 yes 🞎 no
