# Supplementary material for: The effect of chiropractic treatment on infantile colic: study protocol for a single-blind randomized controlled trial
Source: Chiropr Man Therap. 2018 Jun 7;26:17. doi: 10.1186/s12998-018-0188-9 (PMC5991429; doi:10.1186/s12998-018-0188-9)
Supplement: Supplementary file 4 — Consent form. (DOC 34 kb) [file 12998_2018_188_MOESM4_ESM.doc]

**(S5)**

**Samtykke fra forældremyndighedens indehaver til deres barns
deltagelse i et sundhedsvidenskabeligt forskningsprojekt.**

Forskningsprojektets titel: The effect of chiropractic treatment on infantile colic: a RCT

Erklæring fra indehaveren af forældremyndigheden:

Jeg/vi har fået skriftlig og mundtlig information og jeg/vi ved nok om formål, metode, fordele og ulemper
til at give mit/vores samtykke.

Jeg/vi ved, at det er frivilligt at deltage, og at jeg/vi altid kan trække mit/vores samtykke tilbage uden, at min/vores datter/søn mister sine nuværende eller fremtidige rettigheder til behandling.

Jeg/vi giver samtykke til, at ________________________________________(barnets navn)
deltager i forskningsprojektet. Jeg/vi har fået en kopi af dette samtykkeark samt en kopi af den
skriftlige information om projektet til eget brug.

Navnet eller navnene på forældremyndighedens indehaver(e):

____________________________________ _____________________________________

Dato: _______________ Underskrift:

Dato: _______________ Underskrift:

Ønsker du/I at blive informeret om forskningsprojektets resultat samt eventuelle konsekvenser for

Dit/jeres barn?:

Ja____(sæt x) Nej_____(sæt x)

**Erklæring fra den, der afgiver information:**

Jeg erklærer, at forældrene/barnet har modtaget mundtlig og skriftlig information om forsøget.

Efter min overbevisning er der givet tilstrækkelig information til, at forældrene kan træffe beslutning
om barnets deltagelse i forsøget.

Navnet på den, der har afgivet information: Lise Vilstrup Holm

Dato: _______________ Underskrift: _

Projektidentifikation: ( Fx komiteens Projekt-ID, EudraCT nr., versions nr./dato eller lign.)

S-2015001
