# Supplementary material for: The effect of chiropractic treatment on infantile colic: study protocol for a single-blind randomized controlled trial
Source: Chiropr Man Therap. 2018 Jun 7;26:17. doi: 10.1186/s12998-018-0188-9 (PMC5991429; doi:10.1186/s12998-018-0188-9)
Supplement: Supplementary file 5 — Parental diary of infant behavior. (DOCX 17 kb) [file 12998_2018_188_MOESM5_ESM.docx]

**PARENTAL DIARY OF INFANT BEHAVIOR ID.nr.:____**

Four ‘time rulers’ each representing six hours are included on each page and vertical lines indicates fifteen minutes intervals.

|  |  |  |  |  |  |  |  |  |  |  |  |  |  |  |  |  |  |  |  |  |  |  |  |  |  |
| --- | --- | --- | --- | --- | --- | --- | --- | --- | --- | --- | --- | --- | --- | --- | --- | --- | --- | --- | --- | --- | --- | --- | --- | --- | --- |
|  |  |  |  |  |  |  |  |  |  |  |  |  |  |  |  |  |  |  |  | X |  |  |  |  | O |

Sleep Awake*^1^ Crying*^2^ Held/rocked*^3^  Feeding*^4^ Faeces

*^1^ Crying due to e.g. hunger or defecation and easily overcome shall be marked as awake. So crying due to an obvious reason easily comforted shall be marked as awake.

*^2^ Crying due to no obvious reason where the child cannot be comforted by any attempt from the parents.

*^3^ The crying is only partly limited because the child is constantly held/rocked in arm/baby carriage/car etc.

*^4^ Feeding: type of feeding is noted above the line as either breast (B), formula (F) or other fluid such as e.g. chamomile tea (V).

**Eksempel**

|  |  |  | V |  |  |  |  |  |  |  | B |  |  |  |  |
| --- | --- | --- | --- | --- | --- | --- | --- | --- | --- | --- | --- | --- | --- | --- | --- |
|  |  |  |  |  |  |  |  |  |  |  |  |  |  |  |  |
|  |  |  | X |  |  |  |  | O |  |  | X | X |  |  |  |

12.00 13.00 14.00 15.00 16.00

noon

**DATE: _________________**

**Night**

|  |  |  |  |  |  |  |  |  |  |  |  |  |  |  |  |  |  |  |  |  |  |  |  |
| --- | --- | --- | --- | --- | --- | --- | --- | --- | --- | --- | --- | --- | --- | --- | --- | --- | --- | --- | --- | --- | --- | --- | --- |
|  |  |  |  |  |  |  |  |  |  |  |  |  |  |  |  |  |  |  |  |  |  |  |  |

00.00 00.30 1.00 1.30 2.00 3.00 4.00 5.00 6.00

midnight

**Morning**

|  |  |  |  |  |  |  |  |  |  |  |  |  |  |  |  |  |  |  |  |  |  |  |  |
| --- | --- | --- | --- | --- | --- | --- | --- | --- | --- | --- | --- | --- | --- | --- | --- | --- | --- | --- | --- | --- | --- | --- | --- |
|  |  |  |  |  |  |  |  |  |  |  |  |  |  |  |  |  |  |  |  |  |  |  |  |

6.00 7.00 8.00 9.00 10.00 11.00 12.00

noon

**Afternoon**

|  |  |  |  |  |  |  |  |  |  |  |  |  |  |  |  |  |  |  |  |  |  |  |  |
| --- | --- | --- | --- | --- | --- | --- | --- | --- | --- | --- | --- | --- | --- | --- | --- | --- | --- | --- | --- | --- | --- | --- | --- |
|  |  |  |  |  |  |  |  |  |  |  |  |  |  |  |  |  |  |  |  |  |  |  |  |

12.00 13.00 14.00 15.00 16.00 17.00 18.00

noon

**Evening**

|  |  |  |  |  |  |  |  |  |  |  |  |  |  |  |  |  |  |  |  |  |  |  |  |
| --- | --- | --- | --- | --- | --- | --- | --- | --- | --- | --- | --- | --- | --- | --- | --- | --- | --- | --- | --- | --- | --- | --- | --- |
|  |  |  |  |  |  |  |  |  |  |  |  |  |  |  |  |  |  |  |  |  |  |  |  |

18.00 19.00 20.00 21.00 22.00 23.00 24.00

Midnight

|  |
| --- |
|  |
|  |

This was a typical day.

This was not a typical day, because ___________________________________
