# Supplementary material for: The effect of chiropractic treatment on infantile colic: study protocol for a single-blind randomized controlled trial
Source: Chiropr Man Therap. 2018 Jun 7;26:17. doi: 10.1186/s12998-018-0188-9 (PMC5991429; doi:10.1186/s12998-018-0188-9)
Supplement: Supplementary file 6 — Interview-based questionnaire 2. (DOC 28 kb) [file 12998_2018_188_MOESM6_ESM.doc]

Appendix 5:

*Interview 2*

*Filled out by PI at second visit*

**Interview with parents as a supplement for the three days of baseline registration in diary**

1. **Date:** _______________
2. **Idenfication number of child:** _________________
3. **Date of birth for child:** _______________
4. **Start date in the project:** _______________
5. **Development in colic from first interview and until now:**🞎 stopped 🞎 decreased 🞎 unchanged 🞎 increased
6. **Typical number of hours with colic per day:**
7. **If number of hours with colic varies from day to day, state the extremes here:** minimum: maksimum: __________
8. **The colic occurs in so short episodes, so the number of hours is difficult to assess.** 🞎 yes 🞎 no
9. **Typical number of colic episodes per day:**
10. **If number of episodes with colic varies from day to day, state here the extremes**:
     minimum: maximum: __________
11. **Mark the time spans, where the colic normally occurs:**🞎 Kl. 06-12 🞎 Kl. 12-18 🞎 Kl. 18-24 🞎 Kl. 24-06
