# Supplementary material for: The effect of chiropractic treatment on infantile colic: study protocol for a single-blind randomized controlled trial
Source: Chiropr Man Therap. 2018 Jun 7;26:17. doi: 10.1186/s12998-018-0188-9 (PMC5991429; doi:10.1186/s12998-018-0188-9)
Supplement: Supplementary file 7 — Parents final questionnaire. (DOC 35 kb) [file 12998_2018_188_MOESM7_ESM.doc]

**Appendix 6:**

*Questionnaire*

*Filled out by parents at the end of project*

1. **Date:** _______________
2. **Idenfication number of child:** _________________
3. **Date of birth for child:** _______________
4. **Do you believe the child was treated?** 🞎 yes 🞎 no
5. **Is the child more comfortable now than before the project started?** 🞎 yes 🞎 no
6. **Is the child more uncomfortable now than before the project started?** 🞎 yes 🞎 no
7. **How is the colic now, when compared with before start in the project?**🞎 stopped 🞎 decreased 🞎 unchanged 🞎 increased
8. **Mark the time spans, where the colic normally occurs:**🞎 06-12 🞎 12-18 🞎 18-24 🞎 24-06

1. **Has there been any change in the child’s defecation routines, since start in the project?** 🞎 ja 🞎 nej
   1. If yes (only one mark):

The child has more often defacation 🞎

The child has more rarely defacation 🞎

- 1. If yes (only one mark):

It is easier now for the child to defacate 🞎

It is more difficult for the child to defacate….. 🞎

1. **Has there been any change in the child’s burps since start in the project?** 🞎 yes 🞎 no
   1. If yes (only one mark):

It is easier for the child to burp 🞎

It is more difficult for the child to burp 🞎

1. **Has there been any change in the child’s regurgitation since start in the project?** 🞎 yes 🞎 no
   1. If yes (only one mark):

The child regurgitates more 🞎

The child regurgitates less 🞎

1. **Has there been any change in the child’s hiccups since start in the project?** 🞎 yes 🞎 no
   1. If yes (only one mark):

The child has more often hiccups 🞎

The child has less often hiccups 🞎

1. **Have you noticed any other change?**  🞎 yes 🞎 no
   1. If yes, please describe the changes: ______________________________________________________
      ______________________________________________________
      ______________________________________________________
2. **Has the child received other treatment for colic in the project period?**
3. 🞎 yes 🞎 no

a. If yes, specify treatment?_________________________

1. **Has the child received treatment for other problems in project period?**  🞎 yes 🞎 no

a. If yes, specify problems?________________________

a. If yes, specify treatment?_________________________

1. **Are you overall satisfied with participating in the project?** 🞎 yes 🞎 no
2. **Comments/feedback:**

**______________________________________________________________ ______________________________________________________________________________________________________________________________________________________________________________________________________________________________________________________________________________________________________________________________________________________________________________________________________________________________________________________________________________________________________________________________________________________________________________**

1. **E-mail address (voluntary -used for sending study results at the end of project):**

_____________________________________________________________________
